# Broadband noise insulation of windows using coiled-up silencers consisting of coupled tubes

**DOI:** 10.1038/s41598-021-85796-0

**Published:** 2021-03-18

**Authors:** Shuping Wang, Jiancheng Tao, Xiaojun Qiu, Ian S. Burnett

**Affiliations:** 1 Centre for Audio, Acoustics and Vibration, Faculty of Engineering and Information Technology, University of Technology Sydney, Sydney, Australia; 2 Key Laboratory of Modern Acoustics and Institute of Acoustics, Nanjing University, Nanjing, China

**Keywords:** Acoustics, Mechanical engineering

## Abstract

It has been demonstrated that a staggered window achieves better noise reduction performance than a traditional single glazing one at middle to high frequencies while maintaining a degree of natural ventilation. There is, however, little improvement in the low frequency range. In contrast, this work proposes to apply coiled-up silencers consisting of coupled tubes on the side walls of staggered windows to obtain noise attenuation in a broad band, especially in the low frequency range. Each element in the silencer consists of two coupled tubes with different cross sections so that noise at more frequencies can be attenuated than that with a uniform cross section. The simulation results show that 8.8 dB overall insertion loss can be obtained between 100 and 500 Hz after applying a combination of silencers designed at 7 different frequencies, and the insertion loss of the staggered window is increased from 6.7 to 15.6 dBA between 100 and 2000 Hz for normal incident traffic noise with the proposed silencers installed. The design is validated by the experiments with a 1:4 scale down model.

## Introduction

Traffic noise is a significant source of noise pollutions and can have a serious effect on people’s daily lives. The weakest point for noise transmission into buildings are windows which are expected to achieve both noise reduction and natural ventilation simultaneously. A staggered window, where the opening sashes of a double glazing window are staggered, is an improved design of natural ventilation windows. It has been demonstrated that the approach performs better than single glazing windows at middle to high frequencies; however, there is no significant improvement in the low frequency range^1–7^. Due to the resonances of the ventilation duct, the sound pressure levels (SPLs) at some low frequencies are even increased using the staggered window. Active noise control, which performs well at low frequencies can be applied on staggered windows to improve the noise reduction in the low frequency range, but with increased cost and system complexity^8–10^.

Passive reactive silencers, like Helmholtz resonators, plenum chambers and side branch tubes are options to reduce low frequency noise^11–18^. However, the size of the resonator or the length of the side branch tube increases prohibitively at low frequencies, preventing their usage in practical applications. Space-coiling, which has been widely used in the design of ultra-thin acoustic absorbers is an effective way to reduce the size of these structures^19–26^. A sound insulator with subwavelength thickness was previously designed based on space-coiled tubes, and both numerical simulation and experimental results demonstrate its feasibility to reduce noise around 4346 Hz^27^. A hybrid structure designed at 5 different frequencies can extend the working band; however, it only works in the high frequency range and the 200 Hz bandwidth is still too narrow for traffic noise control.

This paper designs a hybrid silencer working in a broad band at low frequencies and applies the solution to a staggered window to create a natural ventilation window that blocks noise across the frequency band from 100 to 2000 Hz. By coupling two tubes with different cross sections and coiling them in space, each silencer element works at more than two frequencies below 500 Hz. The total thickness of the silencer is only 1/10 the wavelength of 100 Hz. A total insertion loss of 8.8 dB can be achieved between 100 and 500 Hz with the hybrid silencer, and the insertion loss of the staggered window with the proposed silencers installed is shown to increase from 6.7 to 15.6 dBA between 100 and 2000 Hz for normal incident traffic noise. Compared with the recent work in noise insulation ventilation windows, which either work in a limited frequency band^28,29^, or in a broad band but at higher frequencies^26,30,31^, this work extends the working frequencies of the silencer to as low as 100 Hz, and increases the effective band width to 1900 Hz. Another improvement is that we carried out experiments with a real scale down model of a staggered window, instead of in an impedance tube with normal incident sound, thus demonstrated the feasibility of the silencer to be used in practical applications.

## Results

**Figure 1:**
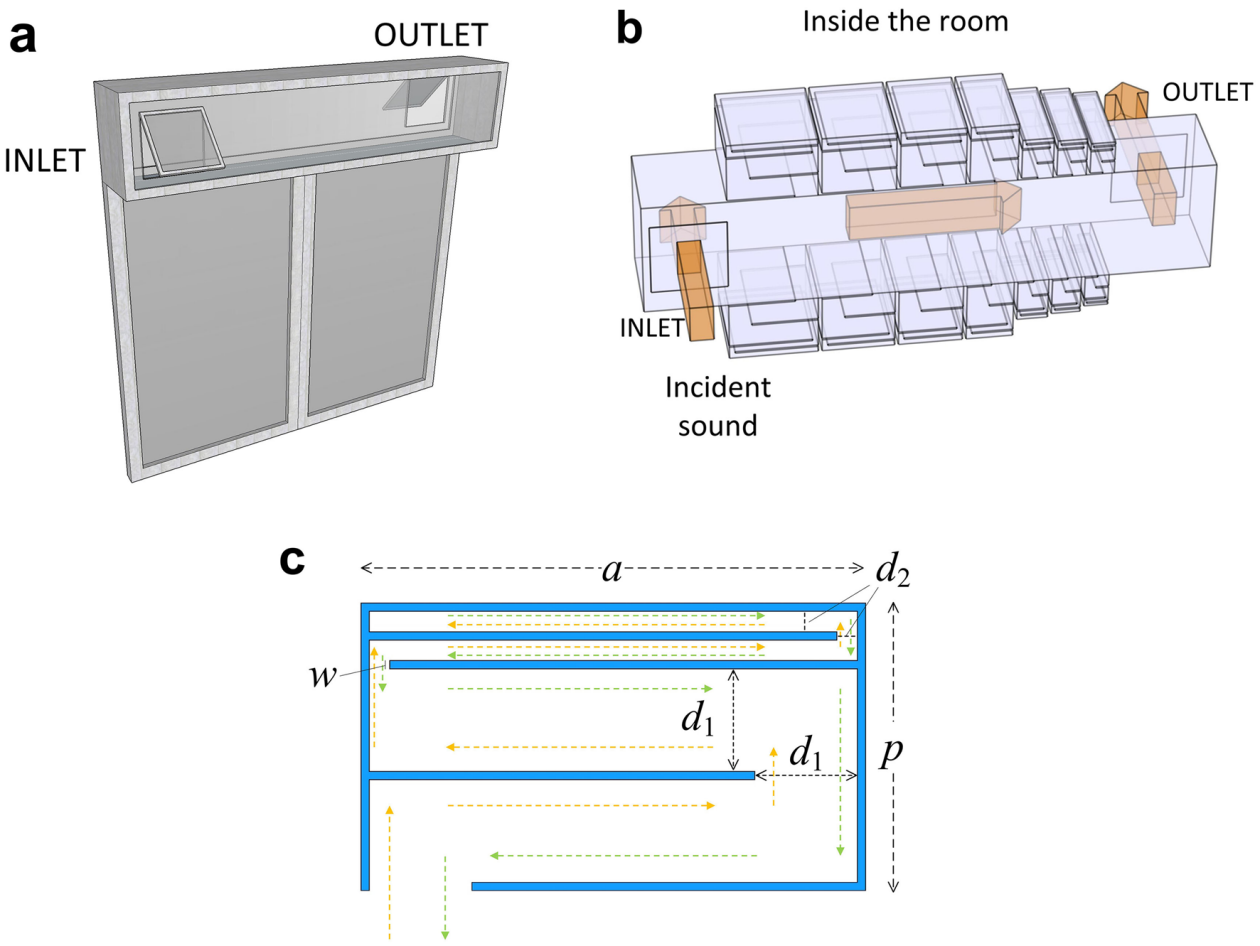
(**a**) A sketch of the staggered window used in practice. (**b**) The ventilation duct of the staggered window with the proposed silencers installed. (**c**) A cross-sectional sketch of each element in the proposed silencer.

A staggered window can be used with a traditional single glazing window, as shown in Fig. 1(a), to provide sufficient natural ventilation while the single glazing window is closed. The silencers we proposed are attached on the two opposite side walls of the ventilation duct of the staggered window, as shown in Fig. 1(b). The outside noise transmits through the inlet into the duct and radiates to the internal room through the outlet. The size of the ventilation duct under investigation is 1.5 m × 0.32 m × 0.25 m (*l*_*x*_ × *l*_*y*_ × *l*_*z*_), and the sizes of the inlet and outlet for ventilation are both 0.2 m × 0.2 m.

For this specific window, the silencer on both sides of the duct consists of 7 elements of different sizes. Figure 1(c) shows a cross-sectional sketch of one element in the silencer. It consists of two tubes, with a cross section of *d*_1_ and *d*_2_, respectively. Both tubes are folded up once in space, and the total size of the silencer is *a* × *p* (width × thickness). In the numerical simulations, the primary sound transmits into the staggered window through the inlet and radiates outside to a semi-infinite space through the outlet. The SPL at an evaluation point outside the outlet 0.1 m away from its center is used to evaluate the performance of the silencers. The noise reduction caused by the silencers is defined as the difference between the SPLs at the evaluation point without and with them installed, and it is also the insertion loss (IL) of the silencers.

**Figure 2:**
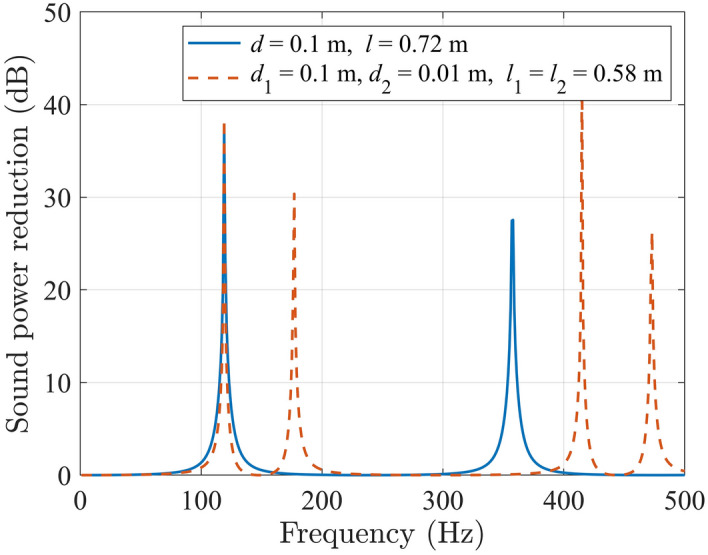
The reduction of the sound power radiated to the downstream in a duct with a side branch tube with a uniform cross section or two coupled tubes with different cross sections.

By using two tubes with different cross sections coupled together and adjusting the lengths and cross sections of the two tubes, the proposed structure works at more frequencies in the low frequency range than that with a uniform cross section. The reduction of the sound power radiated to the downstream when a side branch tube is installed on an infinitely long duct can be predicted theoretically (See “Methods” for more details). As shown in Fig. 2, when the side branch tube has a uniform cross section, and the length is *l* = 0.72 m, the sound power reduction reaches the highest at 119 Hz because a quarter the wavelength of 119 Hz is approximately 0.72 m. There is another sound power reduction peak at 357 Hz, which is the 3rd harmonic of 119 Hz. However, if the side branch tube consists of two parts with different cross sections, it works at 4 frequencies below 500 Hz. Apart from 119 Hz, the reduction at 177 Hz, 415 Hz and 473 Hz are all above 20 dB, indicating that a silencer consisting of two coupled tubes with different cross sections can reduce noise over a wider frequency band. A silencer with coupled tubes might occupy less space than a uniform one in some cases with careful design (for example, in this work a reduction to 0.0159 m^3^ from 0.0180 m^3^ is achieved).

**Figure 3:**
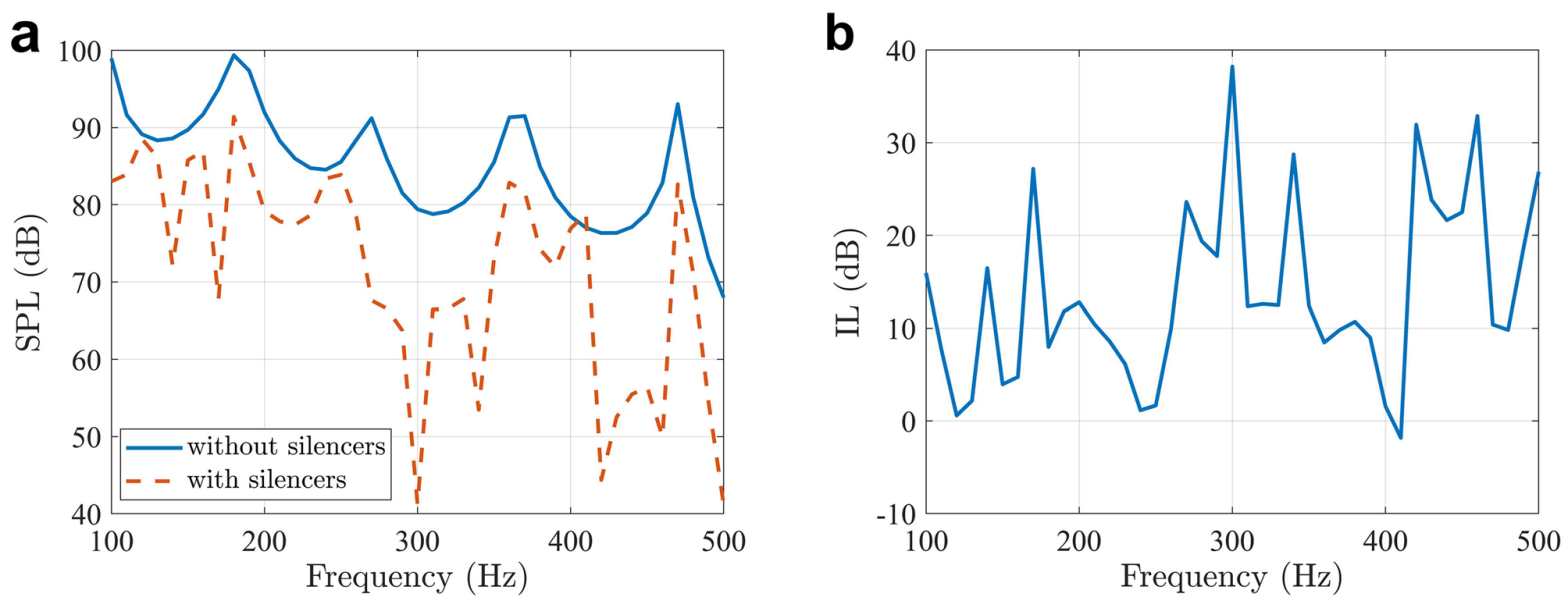
**(a)** The SPLs at the evaluation point with and without the silencers in numerical simulations. **(b)** The insertion loss caused by the silencers.

To reduce the silencer size, the tubes are coiled up in space, as shown in Fig. 1(c). In order to achieve effective broadband noise reduction, silencers with different parameters are arranged in an array along the side walls of the ventilation duct. They are designed to operate at 7 different frequencies, and the parameters are listed in Table. 1. The 7 elements are installed on two opposite sides of the duct, as shown in Fig. 1(b). When the primary source is a normally incident plane wave with a strength of 1 Pa, the SPLs at the evaluation point with and without the silencers installed are shown in Figs. 3(a), and 3(b) shows the insertion loss caused by the silencers.

**Table 1:** The parameters of the 7 silencer elements.

Working frequency (Hz)	*a* (m)	*p* (m)	*d*_1_ (m)	*d*_2_ (m)	*w* (m)	Quantity
130	0.245	0.175	0.060	0.015	0.005	1
160	0.203	0.175	0.060	0.015	0.005	1
182	0.181	0.175	0.060	0.015	0.005	1
255	0.135	0.175	0.060	0.015	0.005	1
375	0.093	0.130	0.040	0.010	0.005	1
395	0.087	0.120	0.036	0.009	0.005	1
475	0.074	0.104	0.030	0.007	0.005	1

Figure 3 shows that noise is reduced at all frequencies between 100 and 500 Hz except at 410 Hz, where the SPL is slightly increased by 1.8 dB. The overall insertion loss caused by the silencers can be used to evaluate the noise reduction performance in a broad band. It is calculated with

1 $${\text{IL}} = 10\log_{10} \left( {\sum\nolimits_{i = 1}^{M} {10^{{\text{SPL}_{{i\_{\text{without}}}} /10}} } } \right) - 10\log_{10} \left( {\sum\nolimits_{i = 1}^{M} {10^{{\text{SPL}_{{i\_{\text{with}}}} /10}} } } \right),$$

where *M* is the number of frequencies of interest, and SPL_*i*_without_ and SPL_*i*_with_ are the SPLs at the *i*th frequency without and with the silencers installed, respectively. It is the reduction of the overall SPL at all the frequencies of interest and the number is 8.8 dB between 100 and 500 Hz.

**Figure 4:**
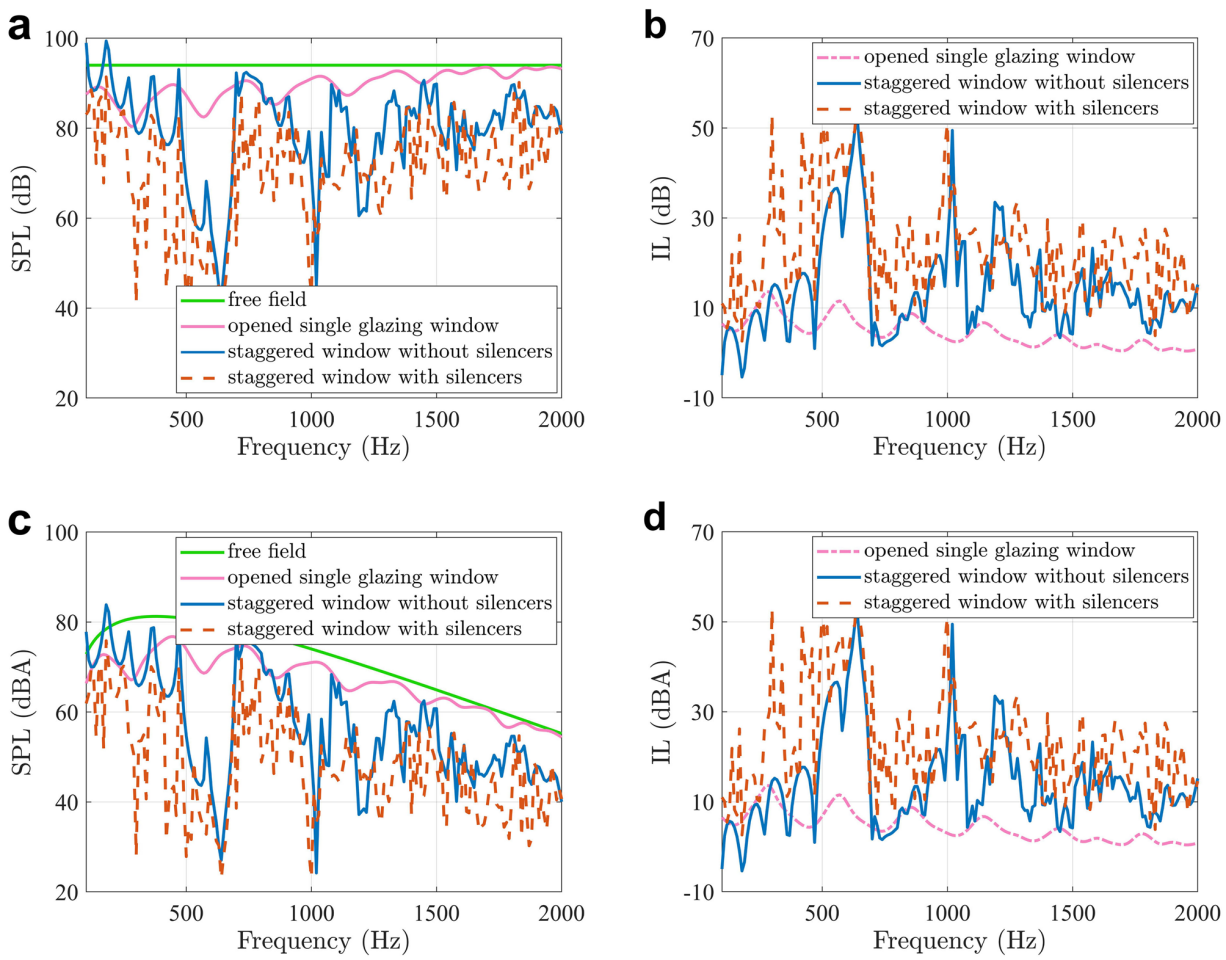
**(a)** The SPLs at the evaluation point, and **(b)** the insertion loss of the windows with normal incident white noise. **(c)** The SPLs at the evaluation point, and **(d)** the insertion loss of the windows with normal incident traffic noise.

The silencers also work at higher frequencies, and Fig. 4(a) shows the SPLs between 100 and 2000 Hz, when the primary sound is a normal incident plane wave with an amplitude of 1 Pa. The blue and red curves correspond to the SPLs outside the staggered window without and with silencers installed, and the SPLs with silencers are lower at 84.3% of the frequencies between 100 and 2000 Hz. The overall insertion loss is 7.2 dB between 100 and 2000 Hz.

The SPLs at the evaluation point in the free field and with an opened single glazing window are also included in Fig. 4(a) for comparison. The single glazing window is embedded in an infinitely large rigid wall. The window size is 0.2 m × 0.2 m, which is the same as the inlet and outlet of the staggered window, and it is kept open to ensure a similar amount of air circulation. The sound field behind the window is different from that in a free field in the low frequency range when the wavelength is comparable to the window size due to the reflections and scattering by the window frame.

It can be observed from Fig. 4(a) and (b) that the staggered window provides extra noise reduction from 500 to 2000 Hz compared with the opened single glazing one, demonstrating the advantage of a staggered window in the middle to high frequency range. The insertion loss of the opened single glazing window is 3.6 dB between 100 and 2000 Hz, and the staggered window increases it to 7.5 dB. By applying silencers on the staggered window, the insertion loss is further increased to 14.7 dB. Figure 4(c) shows the A-weighted SPLs at the evaluation point when the primary sound is normal incident traffic noise, and Fig. 4(d) shows the corresponding insertion loss. The insertion loss of the staggered window with silencers is 15.6 dBA between 100 and 2000 Hz, in comparison to only 6.7 dBA without the silencers.

**Figure 5:**
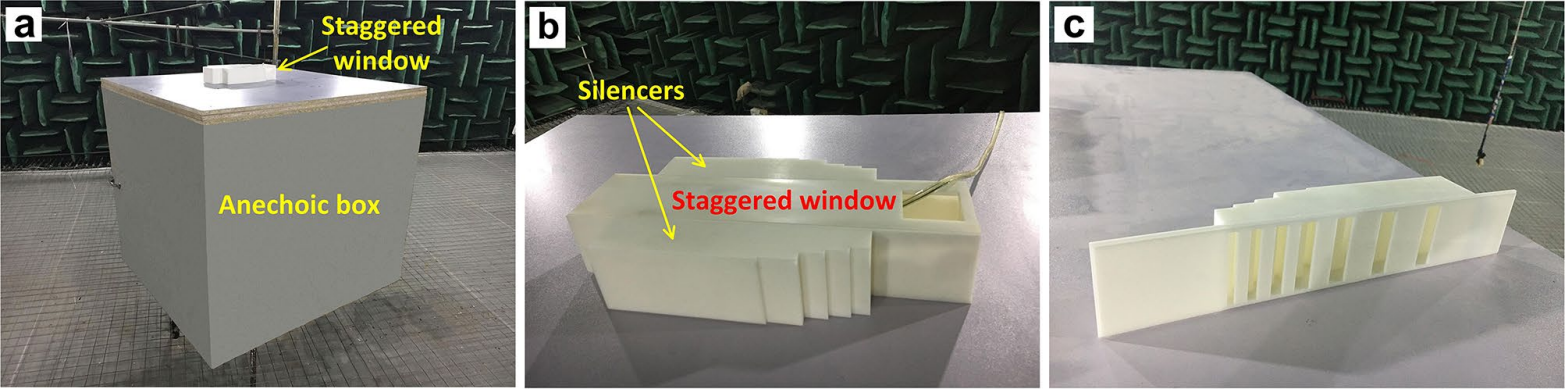
Photos of the experimental setup. (**a**) A panoramic view; (**b**) a detailed view of the staggered window model with silencers installed on the two side walls; and (**c**) the 3D printed silencer.

The experiments were carried out in an anechoic room with a 1:4 scale down model, as shown in Fig. 5(a) and Fig. 5(b) is a detailed view of the staggered window model with the silencers installed. The size of the staggered window model was 375 mm × 80 mm × 67.5 mm, and the size of both the inlet and outlet was 50 mm × 50 mm. The staggered window model was installed at the top of a 1.20 m × 1.10 m × 1.00 m anechoic box, which is a 1:4 scale down model of a rectangular room. The noise source was a loudspeaker inside the box and the silencers were installed on the two opposite side walls of the staggered window model to reduce the noise transmitted through it to the outside.

The silencers were designed for the frequency band from 400 to 2000 Hz for the 1:4 scale model, and the parameters are listed in Table 2. The silencers were built with 3D printing using acrylonitrile-butadience-styrene (ABS) plastic, as shown in Fig. 5(c). Since the density of ABS plastic is 1100 kg/m^3^, the thickness of the walls of the structure was chosen as *w* = 3.00 mm to ensure a transmission loss of at least 20.4 dB at frequencies no less than 400 Hz according to the mass law^32^, thus the boundaries of the structure can be considered as rigid in the experiments at frequencies of interest. The SPL at the point 2 cm above the center of the outlet was measured to evaluate the noise reduction performance of the silencers.

**Table 2:** The parameters of the silencer elements in the experiments.

Working frequency (Hz)	*a* (mm)	*p* (mm)	*d*_1_ (mm)	*d*_2_ (mm)	*w* (mm)
510 Hz	57.40	50.00	15.20	3.80	3.00
620 Hz	47.20	50.00	15.20	3.80	3.00
720 Hz	40.60	50.00	15.20	3.80	3.00
980 Hz	29.30	47.00	14.00	3.50	3.00
1460 Hz	17.90	37.00	10.00	2.50	3.00
1560 Hz	16.40	32.00	8.00	2.00	3.00
1840 Hz	13.20	27.00	6.00	1.50	3.00

**Figure 6:**
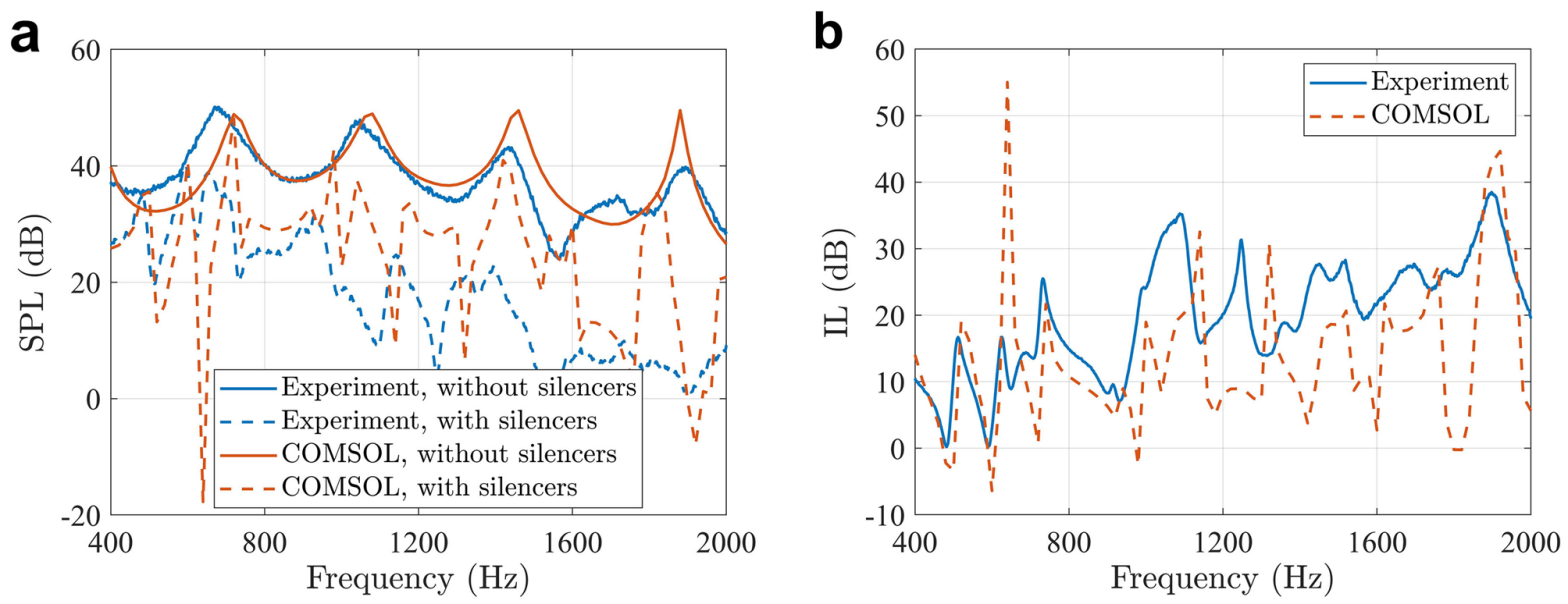
**(a)** The measured SPLs at the evaluation point and the comparisons with the numerical simulation results obtained from COMSOL. **(b)** The insertion loss of the silencers in the experiments and numerical simulations.

The loudspeaker inside the anechoic box generated white noise, and the frequency range of interest was 400 Hz-8000 Hz, corresponding to 100 Hz-2000 Hz for the full scale model. The measured SPLs at the evaluation point with and without the silencers installed are shown in Fig. 6(a), and the insertion loss of the silencers is shown in Fig. 6(b). It is clear that the measured SPL is reduced at most frequencies with the silencers installed, and the overall insertion loss caused by the silencers is 12.0 dB between 400 and 2000 Hz.

The numerical simulation results obtained from the commercial finite element model software COMSOL Multiphysics 5.5 for the same silencer are included in Fig. 6(a) and (b) for comparison. The overall insertion loss caused by the silencers is 8.0 dB within this frequency range in the numerical simulations. The trends of the numerical simulation and experimental results are similar, and one of the reasons for the difference is the errors in 3D printing. Further numerical simulation results (not shown here) indicate that a 0.5 mm difference in the tube width or length can cause a 20 Hz difference in the working frequency, which demonstrates the significant effect of 3D printing errors on the noise reduction performance of the silencer.

Other reasons for the difference between numerical simulation and experimental results include: (1) The model created in COMSOL was not exactly the same as that in the experiments; (2) The primary source in the numerical simulations was a point monopole, while the primary loudspeaker was not omni-directional in the experiments, especially in the high frequency range; (3) The primary loudspeaker was put near a corner of the anechoic box, instead of the position in the numerical simulations which was hard to be installed at. Although there exists difference between numerical simulation and experimental results, the measured results have demonstrated the effectiveness of the proposed silencer.

**Figure 7:**
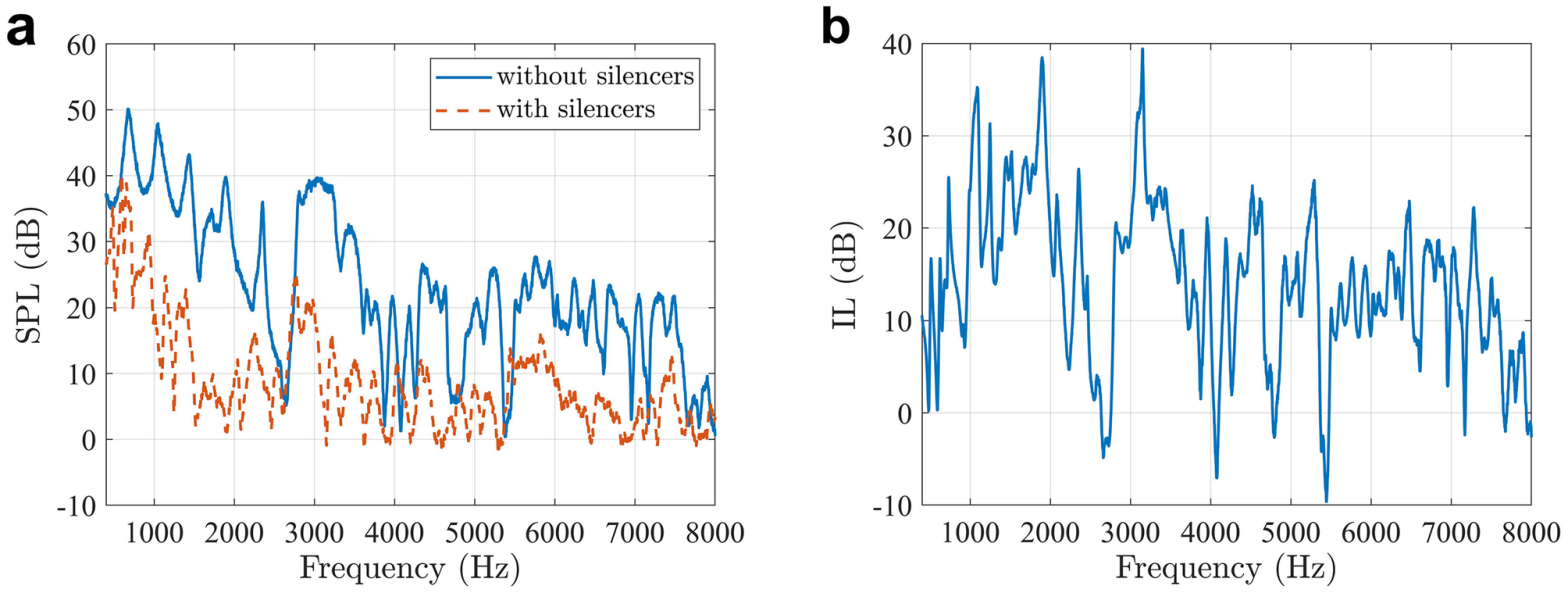
**(a)** The measured SPLs at the evaluation point between 400 and 8000 Hz with and without the silencers. **(b)** The insertion loss of the silencers in the experiments.

The silencers can also reduce noise at higher frequencies, which is shown in Fig. 7(a), contributing to a total insertion loss of 12.6 dB at the evaluation point across the whole frequency range from 400 to 8000 Hz, and the insertion loss spectrum is shown in Fig. 7(b). The experimental results demonstrate that the broadband noise reduction can be achieved by introducing specially designed silencers.

## Discussion

In this paper, we propose applying an array of thin silencers on a staggered window to constitute a natural ventilation window that attenuates noise in a broad frequency band. Each element of the silencer consists of two coupled tubes with different cross sections that are coiled up once in space; this coupled tube operates effectively at two more frequencies than tubes with a uniform cross section. With a hybrid silencer designed at 7 different frequencies, the SPL can be reduced at all frequencies between 100 and 500 Hz, except at 410 Hz, where a slight SPL increase of 1.8 dB is observed. The overall insertion loss of the silencer is 8.8 dB within this frequency range. The thickness of the whole structure is only 1/10 the wavelength of 100 Hz. The silencer also reduces noise at higher frequencies, and the insertion loss of the staggered window between 100 and 2000 Hz can be increased from 6.7 to 15.6 dBA for normal incident traffic noise with the silencers installed. The experimental results achieved with a 1:4 scale down model in the anechoic room show that the overall SPL at a point near the outlet was reduced by 12.6 dB in the frequency range between 400 and 8000 Hz. Both the simulation and experimental results demonstrate the feasibility of broadband noise control with specially designed silencers.

## Methods

### Theory

**Figure 8:**
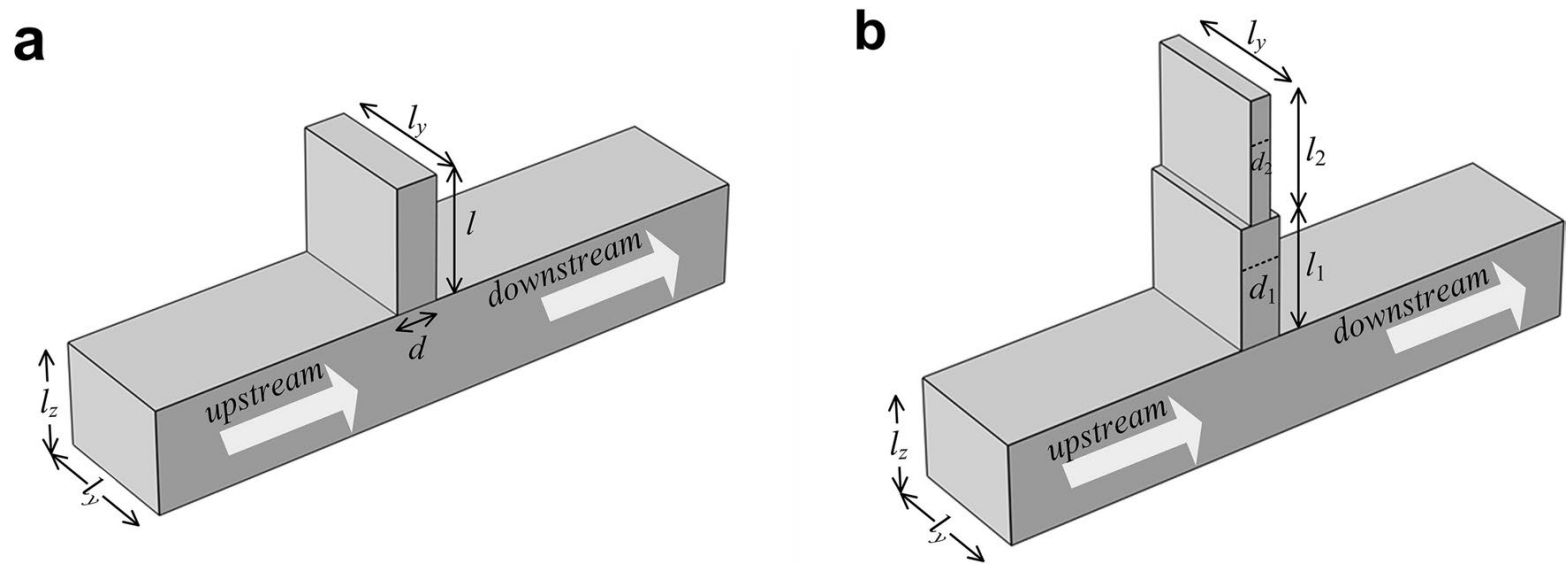
A sketch of the side branch tube in an infinitely long rectangular duct with (**a**) a uniform cross section and (**b**) two different cross sections.

The reduction of the sound power radiated to the downstream of an infinitely long duct can be predicted theoretically. Figure 8 shows the sketches of a duct with two kinds of side branch tubes.

A plane wave is assumed to propagate in the infinitely long duct with a cross section of *S* = *l*_*y*_ × *l*_*z*_, and a side branch tube is attached on the side wall. The acoustic impedance of the side branch tube in Fig. 8(b) is^32^

2 $$Z_{{\text{b}}} = \frac{{\rho_{0} c_{0} }}{{S_{\text{b1}} }}\frac{{Z_{{{\text{b2}}}} + {\text{j}}\frac{{\rho_{0} c_{0} }}{{S_{{\text{b1}}} }}\tan (kl_{\text{1}} )}}{{\frac{{\rho_{0} c_{0} }}{{S_{{\text{b1}}} }} + {\text{j}}Z_{{{\text{b2}}}} \tan (kl_{\text{1}} )}},$$

where *S*_b1_ = *d*_1_ × *l*_*y*_ is the cross section of the side branch tube entrance, *k* is the wavenumber, *ρ*_0_ and *c*_0_ are the air density and sound speed in the air, respectively, and

3 $$Z_{{{\text{b}}2}} = - {\text{j}}\frac{{\rho_{0} c_{0} }}{{S_{{\text{b2}}} }}\cot (kl_{\text{2}} )$$

is the acoustic impedance of the tube with length *l*_2_ in Fig. 8(b). The transmission coefficient of the sound power to the downstream is

4 $$t_{\text{p}} = \frac{{R_{\text{b}}^{2} + X_{\text{b}}^{2} }}{{\left( {\frac{{\rho_{0} c_{0} }}{2S} + R_{\text{b}} } \right)^{2} + X_{\text{b}}^{2} }},$$

where *R*_b_ and *X*_b_ are the real and imaginary parts of *Z*_b_, respectively. The reduction of the sound power is defined as

5 $${\text{NR}}_{\text{p}} = - 10\log_{10} (t_{\text{p}} ).$$

For the side branch tube with a uniform cross section shown in Fig. 8(a), Eq. (2) can be simplified as

6 $$Z_{\text{b}} = - {\text{j}}\frac{{\rho_{0} c_{0} }}{{S_{\text{b}} }}\cot (kl),$$

where *S*_b_ = *d* × *l*_*y*_ is the cross section of the side branch tube entrance. Equation (5) is also used to calculate the sound power reduction in this case.

For coiled-up tubes investigated in this paper, the *l*_1_ and *l*_2_ are the equivalent lengths of the two parts, which are approximately 2*a* − *d*_1_ and 2*a* − *d*_2_, respectively. Applying symmetric silencers on two opposite sides of the ventilation duct increases the noise reduction, but has limited effect on the working frequencies for the case we investigated.

### Numerical simulations

The results in Figs. 3 and 4 for a normal incident plane wave are obtained using LMS Virtual.Lab 13.10. The results in Fig. 6 with a point monopole as the primary sound source are obtained using COMSOL Multiphysics 5.5. The air density is 1.225 kg/m^3^ and the sound speed in the air is 343.202 m/s. All the boundaries of the models used in COMSOL and Virtual.Lab are set as rigid.
